# Chronotype assessment via a large scale socio-demographic survey favours yearlong Standard time over Daylight Saving Time in central Europe

**DOI:** 10.1038/s41598-020-58413-9

**Published:** 2020-01-29

**Authors:** Martin Sládek, Michaela Kudrnáčová Röschová, Věra Adámková, Dana Hamplová, Alena Sumová

**Affiliations:** 1 Institute of Physiology, the Czech Academy of Sciences, Prague, Czech Republic; 2 Institute of Sociology, the Czech Academy of Sciences, Prague, Czech Republic; 3 Institute for Clinical and Experimental Medicine, Prague, Czech Republic

**Keywords:** Neurophysiology, Medical research

## Abstract

Abandoning daylight saving time in Europe raises the topical issue of proper setting of yearlong social time, which needs mapping of various socio-demographic factors, including chronotype, in specific geographic regions. This study represents the first detailed large scale chronotyping in the Czech Republic based on data collected in the complex panel socio-demographic survey in households (total 8760 respondents) and the socio-physiological survey, in which chronotyped participants also provided blood samples (n = 1107). Chronotype assessment based on sleep phase (MCTQ questions and/or time-use diary) correlated with a self-assessed interval of best alertness. The mean chronotype of the Czech population defined as mid sleep phase (MSFsc) was 3.13 ± 0.02 h. Chronotype exhibited significant east-to-westward, north-to-southward, and settlement size-dependent gradients and was associated with age, sex, partnership, and time spent outdoors as previously demonstrated. Moreover, for subjects younger than 40 years, childcare was highly associated with earlier chronotype, while dog care was associated with later chronotype. Body mass index correlated with later chronotype in women whose extreme chronotype was also associated with lower plasma levels of protective HDL cholesterol. Based on the chronotype prevalence the results favour yearlong Standard Time as the best choice for this geographic region.

## Introduction

The circadian system of human beings controls processes which keep them behaviourally active predominantly during the daylight. This is achieved through synchronization (entrainment) of their endogenous circadian (from Latin *circa-dies*; about a day) system with 24 h solar cycles of alternation in light and darkness. In principle, light intruding into the evening hours delays faster clocks and light intruding into the morning hours advances slower clocks, resulting in entrainment to a 24 h day^1–3^. However, individual subjects vary greatly in the exact time when during the day they feel more capable of performing specific tasks and when they prefer to sleep. This produces a tendency to shift preferable activity/sleep schedules relative to the solar time. The phenomenon is called chronotype. When first introduced, the term chronotype was perceived as a personal trait that defined individual temporal phenotype^4^. This concept is supported by the fact that it can be tracked not only in humans living in modern society, but also in cultures with pre-industrial lifestyles^5^, and even in animal species^6,7^. Chronotype is now often considered as a complex concept reflecting expression of internal clock’s phase in real life conditions (for review, see^8^). Regardless of definition, chronotype exhibits almost Gaussian distribution in population^9^, spanning across many hours between the extremes, so that in conditions when the extreme chronotypes are free to organize their activity and sleep independent of social constrains, their active part of day may shift well into the nighttime hours.

The mechanisms determining chronotype are not well understood. A most plausible explanation for the underlying mechanism is that inter-individual variation among endogenous circadian periods occurs within a population^10–14^. In case of a weak synchronizing cue this factor affects the phase angle of entrainment of the clocks in our body^15^; a longer period leads to tendency towards a later type and shorter period towards an earlier type. The variation in circadian periods in population is based on genetic variability in the mechanisms that fine-tune the molecular core clockwork^16–18^ (for review, see^19^). However, factors that determine chronotype remain rather elusive, because it changes remarkably depending on many, rather independent, variables. Heritability of 40–60% is reported based on most studies on twins (reviewed in^20^) as well as recent genome-wide association studies (reviewed in^21^). Cross-sectional population studies^9,22^ as well as rare longitudinal studies^23,24^ show that chronotype changes throughout lifespan, being earlier in childhood, latest during adolescence (up to about 20 years old), and earlier again during adulthood to elderly. Interestingly, on the individual level, early chronotypes exhibit higher persistence of their diurnal preferences with aging^24^. Chronotype may also depend on sex, because men tend to be later chronotypes than women^25,26^, though this depends on the selected population sample^27^. Finally, both parameters, age and sex, may interrelate in modulating chronotype^28^. Importantly, chronotype can be influenced also by various external factors. Therefore, it remains to be elucidated whether and how these factors interact with circadian clock during the lifespan in humans.

The dominant external factor that determines chronotype is the degree of synchrony between endogenous clocks and solar cycle. It is obvious that expression of chronotype is amplified by a weak strength of solar cycle to entrain the clock, which then tends to drift according to its internal period. This typically occurs in people living in modern industrial society favouring prevalently indoor activities (accompanied with low daylight exposure), often with a tendency to its extension into the nighttime hours (accompanied with exposure to artificial light at night). Both factors reduce amplitude of the natural light/dark signal (solar cycle) needed to entrain the clock. Consequently, in extreme chronotypes the combination of a free choice in organizing daily schedule, along with poor entraining cues, leads to abnormal phase-angle of clock entrainment with the solar cycle. Importantly, the late chronotypes are actually able to entrain their clocks as demonstrated in subjects, whose clocks and sleep/wake cycles were delayed in urban environment, but advanced upon their exposure to full-spectrum natural daylight and nights without any artificial light^29,30^. Experimental phase advancement of clock in late chronotypes had positive effect of their mental health and performance^31^. Nevertheless, the original preference of sleep phase returned back when the subjects were released from the entrainment^32^. Whether this applies also to chronotype defined as a personal trait is not clear.

For synchronizing activity among its members, human society uses social time, which is to some extent derived from the solar cycle and geographic location. When extreme chronotypes with weakly entrained clocks are forced to adapt their activity/working hours according to the social time, and are active during their subjective night, they may experience social jet lag, a term introduced by Till Roenneberg in 2006^33^ for various symptoms caused by misalignment between internal and social time. Importantly, the individual’s chronotype is related to phase not only of the central, but also the peripheral, clocks^34^ so that they all may become misaligned with the social time with complex consequences for our health (for example, see^35^). Therefore, to maintain internal clocks of extreme chronotypes properly synchronized with the social time, they need avoid exposure to artificial light late into the night hours and expose themselves sufficiently to outdoor daylight. This requires proper setting of social time with respect to the geographic location, that is, the relevant time zone^8,25^.

Annual switching between standard time (ST) and daylight saving time (DST) was introduced in relatively modern history to shift the social time relative to the solar cycle during the summer (currently, from April to October). This is planned to be abandoned in Europe starting 2021 which has lead to a debate on which of these two social times should be maintained yearlong. The decision is dependent highly on geographic location and current setting of the ST. For countries, in which the ST is set according to relevant astronomical time (sun at the highest position at noon), keeping the ST yearlong would maintain the symmetry, although the early dawn on the longest summer days may not be aligned with the beginning in activity of most people in population. Maintaining the DST yearlong means that due to the long photoperiod in summer, daylight would be extended into the evening without delaying darkness in the morning hours when most people are awaken. However, if the DST were maintained into the short winter days, the advanced social time would significantly extend number of days when most people wake up and start working in darkness. The morning light is crucial to maintain synchrony with the solar and social time for all people, but mostly for late chronotypes, and especially the extremes, who need morning light to advance their delayed clocks. The lack of synchrony could potentially have harmful impact on their health^36^. Therefore, information about frequency of chronotypes in specific populations living at various geographic locations, and its relationship with their social and health wellbeing are of utmost importance for knowledge-based decisions on general societal issues.

The results of this study are based on data obtained from residents of the Czech Republic. The country is located at the eastern to central position of the CET zone and represents a relatively homogenous population (population size 10.58 million), which developed its modern/industrial lifestyle historically together with neighbouring German-speaking countries, such as Austria and Germany. We hypothesized that due to the geographic position and historically adopted social time (CET), which almost exactly matches the astronomical time, chronotype in the Czech population will be distributed normally with a peak slightly earlier than that reported in the west-European populations. This finding would favour the yearlong ST to DST because advancing the social time in winter would cause longer exposure of population majority to darkness in the morning, which might worsen synchrony of their clocks with the solar/social time, and lead to higher prevalence of extremely late chronotypes in the population. Therefore, we analysed association of the extreme chronotype with markers of metabolic state to understand the plausible impact of the change to yearlong DST on general health. Apart from chronotyping, we also analysed association between extreme chronotype and selected external factors relevant for social life in the Czech Republic. We believe that these data may contribute to clarification of healthy lifestyle recommendations with regard to the daily activity schedules relative to social time.

## Methods

### Ethical considerations

The study followed the principles of the Declaration of Helsinki and was approved by Ethics Committee of the Institute for Clinical and Experimental Medicine and Thomayer Hospital in Prague (study number G-16–05–02). Written informed consent was obtained from each participant who provided blood samples prior to enrolment in the study after explanation of the study procedures.

### Study participants and data collection

Data were collected by MEDIAN, STEM/MARK and CVVM agencies within two surveys.

Survey I – Czech Household Panel Survey (CHPS) was a representative survey of the Czech non-institutionalized individuals aged 10+ living in private households. Four waves were conducted between 2015 and 2018 and for this study, only data from Wave 1 and Wave 4 were used. In the Wave 1, 5159 households were interviewed in 2015 (between July and November 2015). Within the Wave 4, 3188 households were interviewed in 2018 (between June and October).

Survey II – The socio-physiological survey was conducted between September 2016 and April 2017 within the program “QUALITAS – Wellbeing in health and disease” of the AV21 Strategy of the Czech Academy of Sciences. It employed subjects aged 18+ (n = 1107) who were residents of two cities in the Czech Republic, capital city (Praha) and a smaller town (České Budějovice) including surrounding villages. They were selected for participation using the age, sex, and education quotas. The participants of this survey also provided blood samples (for details, see Blood samples collection below).

### Surveys’ structures and instruments

The questionnaire containing Czech translation of Munich ChronoType Questionnaire (MCTQ) (for details, see Data analyses below) was used in Survey I (Wave 4) and Survey II.

The questions on self-assessment on interval during which the responders feel the highest cognitive alertness (for details, see Data Analyses below) were used in Survey I (Wave 4).

The time-use diary data referring to the activities of the previous day were analysed from Survey I. Only data from Wave 1 was used allowing us to include reasonable group of 10+ years old subjects (their number dropped significantly in later Waves). The time-use diary was a self-completion pen and pencil questionnaire (SAQ) and had two versions; one version was administered to adults, one version to young people aged 10–17. Both versions consisted of 30-minutes slots covering the 24-hour period between 6:00 a.m. of the previous day and 6:00 a.m. of the present day. For each slot, respondents were instructed to choose from a list of activities what describes the best they did. For each time slot, multiple activities could be reported but the diary did not distinguish between the primary and secondary activity. The main difference between the children and adult versions of diary was that both groups selected from different activities. Furthermore, respondents were asked whether it was a typical day or not.

Analyses of binary categories: Data from Survey I were used for analyses of association between chronotype and all factors but blood biomarkers, which were analysed for the Survey II.

### Blood samples collection

The subjects participating in the Survey II provided fasted a single blood sample; the samples were collected from all subjects between September 2016 to June 2017 and high density lipoprotein (HDL) and low density lipoprotein (LDL) cholesterol were measured in two certified laboratories (Synlab, Czech Republic) using a standardized clinical methods (levels are expressed in mmol/l). Only subjects from the capital city Prague (n = 589) and the rural region of České Budějovice and surrounding villages (n = 518) recruited for the pilot survey participated in the blood sampling.

### Data analyses

#### Chronotype assessment

Data based on time-use diary included 8759 subjects aged 10+, however, only subjects who reported that timing of sleep they provided represented their “regular” day were included for estimation of MSF (mid sleep phase on free days) (n = 1429) and MSW (mid sleep phase on work days) (n = 2654). Questions from MCTQ^37^ translated into the Czech language were answered by 5132 subjects aged 18+. MSFsc (mid sleep phase on free days, sleep debt corrected, expressed in hours centred on midnight) was calculated for subjects who reported that they don’t need alarm clock as they can freely arrange their sleep time on free days, or wake up regularly without or before alarm clock during their work days (n = 3277). Afterwards, age and sex-corrected MSFsasc (“normalized chronotype”, a hypothetical chronotype at the age of 30) was additionally computed for each subject by using Prism 8 (Graphpad, USA) to fit separate exponential decay curves to previously calculated MSFsc data for men and for women aged 18+ and normalizing to age = 30 according to^38^ (Y = (Y0 - Plateau)^(-K*X)^ + Plateau; men constants: Y0 = 9.62, Plateau = 2.829, K = 0.067; women constants: Y0 = 10.69, Plateau = 2.947, K = 0.089). Subjects from the lowest and highest MSFsasc deciles were categorized as “Extremely early” and “Extremely late” chronotypes (extremes), respectively, and the remaining 8 deciles were categorized as “Non-extreme” chronotype, which involved slightly early, intermediate and slightly late chronotypes.

Apart from using sleep phase, chronotype was determined based on self-assessment, asking the subjects to report on interval during which they feel the highest cognitive alertness. This interval was used to compute BAmid (“best alertness midpoint”) in hours (n = 5035).

Both instruments from Survey I (MCTQ and BAmid) were collected at different calendar months (between June and October) and seasonal effect on chronotype assessment has been previously reported^39^. Therefore, we tested the MCTQ and BAmid for correlation with photoperiod at which chronotyping was performed (data not shown). No significant effect on MSFsc (assessed by MCTQ), MSF or MSW (both assessed by diary) values collected during different months were found (ANOVA P = 0.09, P = 0.58, P = 0.09, respectively).

#### Formulation and categorization of non-binary questions

Data were obtained from Survey I. Smoking category – “non-smokers” (those that have never smoked or smoked before, but quit), “smokers” (smoke occasionally, 1–5 cigarette/day or more often). Alcohol amount category – “low” (never drink, drink 2 or less alcoholic beverages such as 0.5 l of beer, 0.2 l of wine or 0.05 l of distillate on a single occasion), “high” (drink between 3 to 10, or 11+ alcoholic beverages on a single occasion). Fruit/vegetables consumption category – “often” (eat fresh fruits or vegetables daily or more often), “rarely” (several times per week, weekly, monthly or less often). BMI was calculated based on the reported subjects’ current height and weight.

### Statistical analyses

Data were analysed using Python pandas^40^, numpy^41^, statsmodels^42^, scikit_posthocs^43^ and scipy.stats^44^ libraries and visualized in seaborn^45^ and matplotlib^46^. Student t test or one-way ANOVA (scipy.stats) with Tukey’s post-hoc multiple comparison test (scikit_posthocs) were used to compare groups, Pearson’s correlation (scipy.stats) to analyse linear relationships. Chronotype/age nonlinear relationships were visualized by 4^th^ order polynomial curves fitted using seaborn x_estimator function to calculate means and standard deviations in a set amount of bins (for respective numbers of bins, see figure legends). Histogram were binned according to Fredman-Diakonis rule and fitted with a kernel density estimate (KDE) curve using seaborn.

## Results and Discussion

### Chronotype measures

Several instruments to characterize chronotype have been introduced and widely used for decades (for review, see^8,47^). For purpose of the large-scale surveys, questionnaires represent the best chronotyping tool. There are two mainly used questionnaires, Morningness-Eveningness Questionnaire (MEQ)^48^ and Munich ChronoType Questionnaire (MCTQ)^15^, each of which probes the concept of chronotype from different point. Nevertheless, chronotype assessed by MCTQ and MEQ correlate^49^. For purpose of our goal, MCTQ was selected as the tool of choice because it provides information on the sleep phase position for both free days and working days and allows thus assessment of distribution of the sleep phase within the population. Using the questions, chronotype was determined as a sleep debt-corrected mid sleep phase on free days, MSFsc. Apart from MCTQ, for chronotype assessment we used a marker reflecting subjective perception of individual’s chronotype. The subjects reported the time interval of their highest subjective alertness during the day, from which we calculated its midpoint (Best Alertness interval midpoint, BAmid).

**Figure 1:**
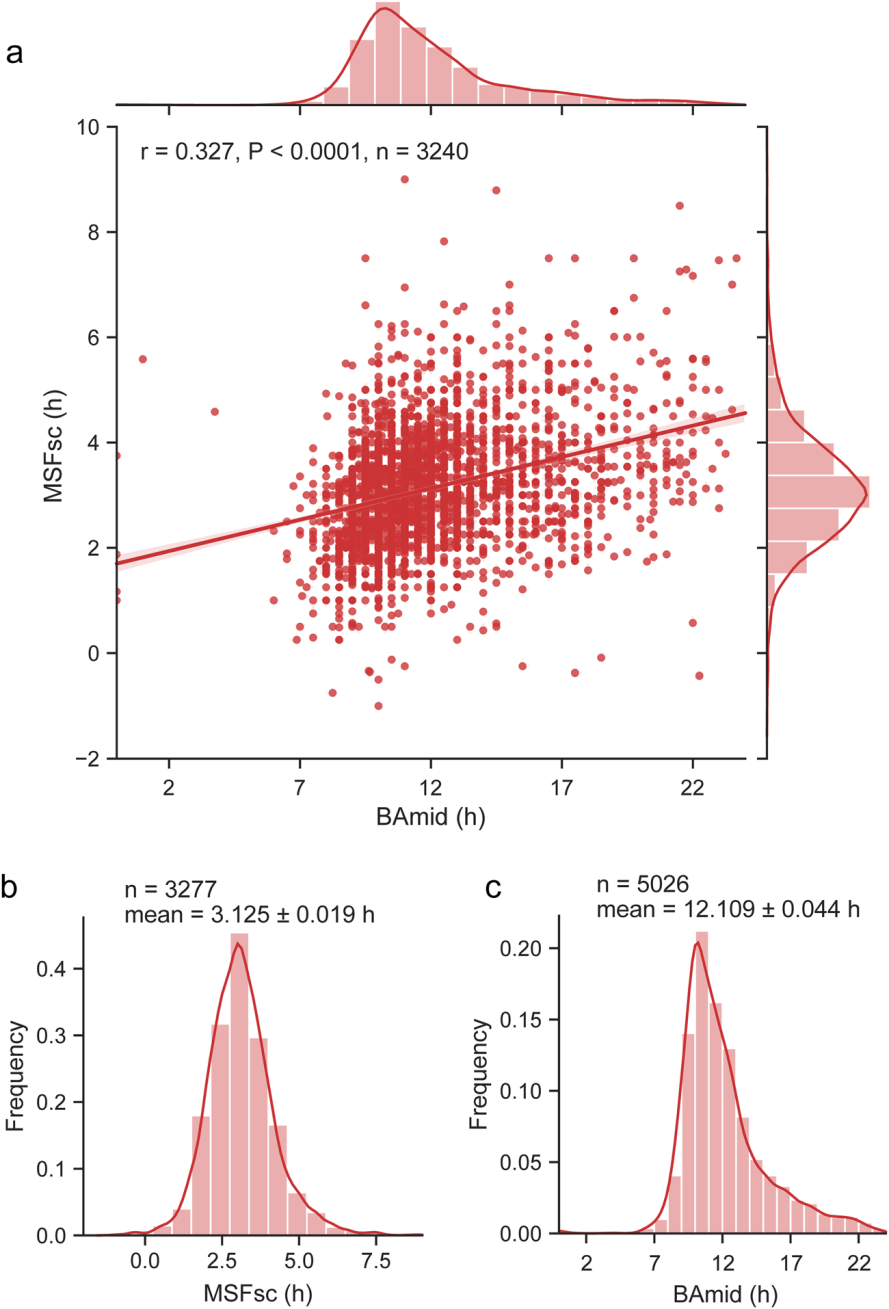
Chronotype distribution in the Czech Republic. (**a)** Chronotypes assessed as MSFsc (in hours centred on midnight) and BAmid (in hours) are significantly positively correlated. (**b)** Population frequency distribution histogram with kernel density estimate (KDE histogram) of MSFsc, (**c**) KDE histogram for BAmid. Pearson correlation coefficient (r) and its P value, n is the number of subjects used to calculate chronotype.

Chronotype was possible to reliably calculate only for part of the survey participants, either due to reporting irregular sleep schedules (which would distort the MSFsc calculation) or inability to self-assess BAmid for various reasons. Finally, chronotype was calculated using MSFsc in 3270 subjects and using BAmid in 5026 subjects. The results revealed a highly significant positive correlation between chronotype assessed by both parameters (n = 3240; Pearson correlation coefficient r = 0.327; P < 0.0001) (Fig. 1a), confirming relevance of data and methodology used to assess chronotype in this study.

### Chronotype distribution

Chronotype defined as MSFsc exhibited normal distribution with a mean value at 3.125 h ± 0.019 (SEM) (SD = 1.0844) (Fig. 1b). Recent studies employing large sample size from countries all over the world (n = ≈ 300000) detected mean MSFsc at about the same time or only slightly later when compared with our dataset (for review, see^8^). Chronotype defined as BAmid had a mean at 12.109 h ± 0.044 (SEM) (SD = 3.1410) and exhibited a slightly asymmetric distribution skewed to the later types (Fig. 1c). The results show that although MSFsc and BAmid do correlate (Fig. 1a), their distribution in population is not identical; either self-assessment of the highest alertness overestimates the late chronotype, or assessment of the sleep phase is underscoring the late chronotype. Therefore, for most of the further analyses, we used both methods of chronotype assessment.

### Geographic and settlement size

**Figure 2:**
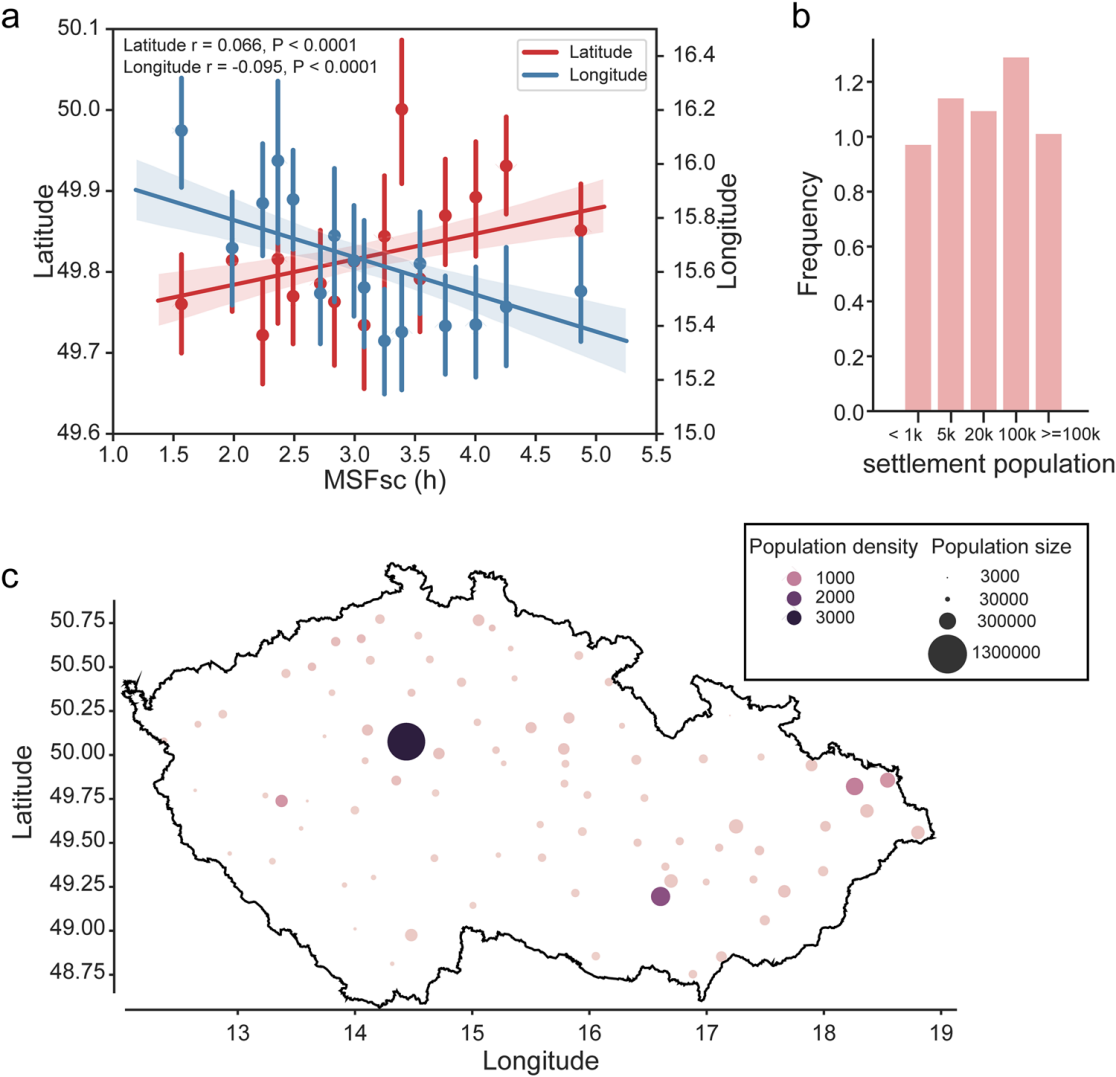
Latitudinal and longitudinal cline and chronotype. (**a**) Latitude (left; red), longitude (right; blue) were plotted against MSFsc to show their effect on chronotype. Means ± SD were calculated using x_estimator function in seaborn Python library in 16 MSFsc bins and analysed by linear regression (line with shading denoting a confidence interval computed by inbuilt bootstrap procedure, r and P values show the result of Pearson correlation). (**b**) Frequency histogram of settlement sizes included in the survey. (**c**) Scatter plot of approximate GPS coordinates of all subjects with calculated MSFsc values. Settlement size coded as point size, local population density coded as colour hue, black contour shows the state borders.

**Figure 3:**
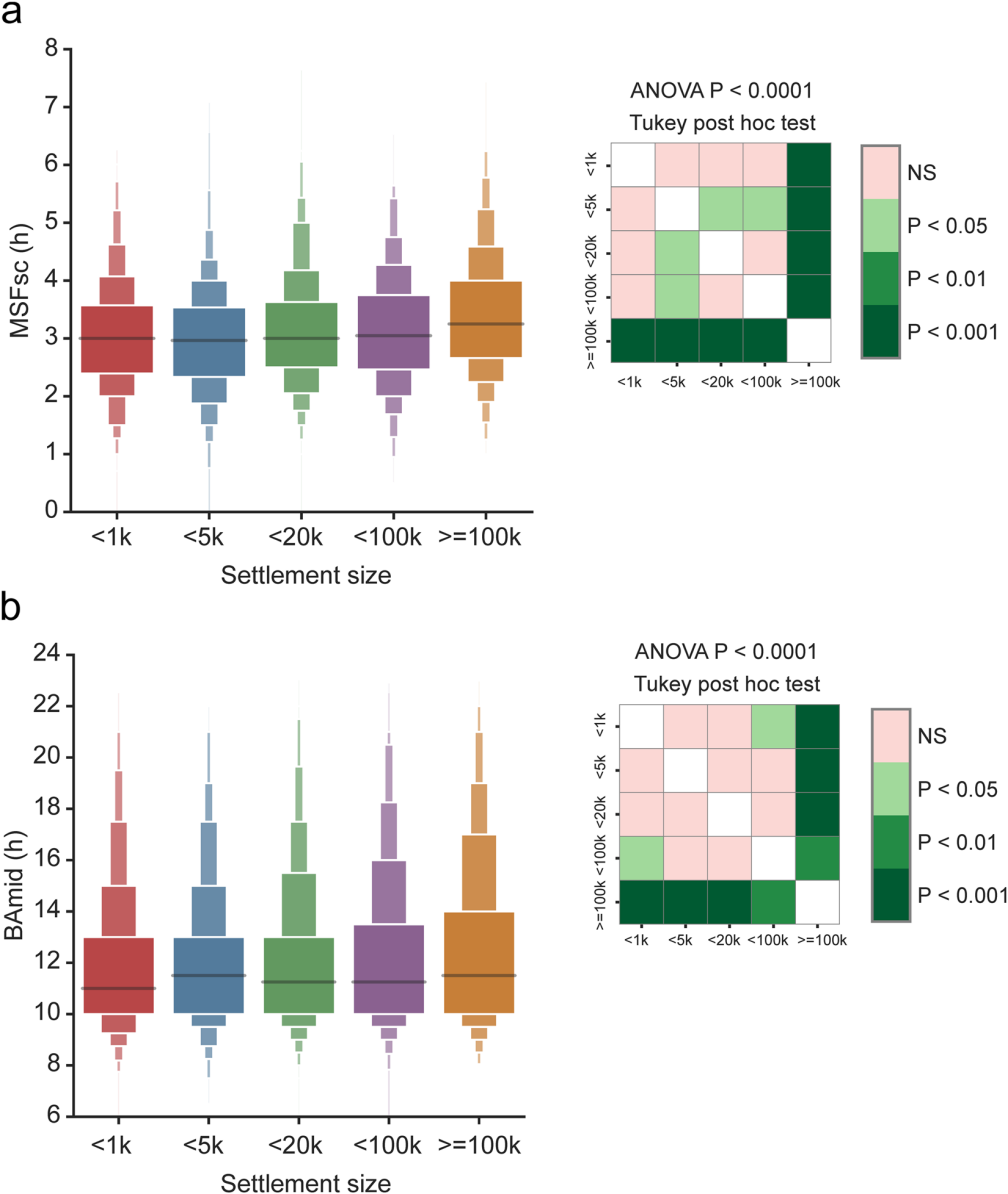
Settlement size and chronotype. (**a**) Boxen plot that shows distribution in quantiles (letter values) of MSFsc categorized according to population in the place of subject’s residence. (**b**) Boxen plot of BAmid categorized as in a. Both MSFsc and BAmid are significantly associated with settlement size as shown by one way ANOVA. Heat maps show results of Tukey post hoc multiple comparison test; k is the shorthand for thousands.

Analyses of the association between geographic location and MSFsc (Fig. 2a) confirmed significant effect for latitude (Pearson’s r = 0.066, P < 0.0001), with north-to-south gradient correlating to later-to-earlier chronotype gradient, and effect for longitude (r = −0.095, P < 0.0001), with east-to-west gradient correlating to the earlier-to-later chronotype. The data provide evidence that geographic location and chronotype correlate even within the relatively small geographic area (Czech Republic, 78 866 square km; 49°45′N 15°30′E). The gradients most likely reflect the difference in timing of sunrise, which on average differs by 25 min between the westernmost and easternmost locations and by 10 min between the northernmost and southernmost locations (https://www.suncalc.org/). However, it also could be related to other factors. One of them is the size of the settlement and their geographic distribution within the country. The urban/rural settlement and population density, as well as location of larger cities, are not absolutely equally distributed between the east and west part of the country (Fig. 2b,c). Chronotype of respondents living in villages/towns/cities of various sizes correlated with the settlement size when assessed as MSFsc (Fig. 3a) or BAmid (Fig. 3b), with later chronotype being more prevalent in larger settlements (ANOVA P < 0.0001). This result is in accordance with differences in light exposure and life style dependent of the city size. In larger cities, exposure to artificial light at night (level of light pollution) is higher on average than in smaller settlements^50^. Also, working hours start typically later due to a higher proportion of business and administrative offices compared to mostly rural employment in smaller settlements. The settlement size is tightly interrelated with other social and life style factors, which may also participate in the observed effects on geographic chronotype distribution (for more data, see below the Lifestyle factors). These results demonstrate that in our population sample both the geographic location and size of the settlement correlate with the prevalent chronotype when assessed as sleep phase, as well as self-perceived feeling of alertness. This is in accordance with previous observations that sleep phase in more rural areas is earlier than in more urban areas^38,51^.

### Age and sex

**Figure 4:**
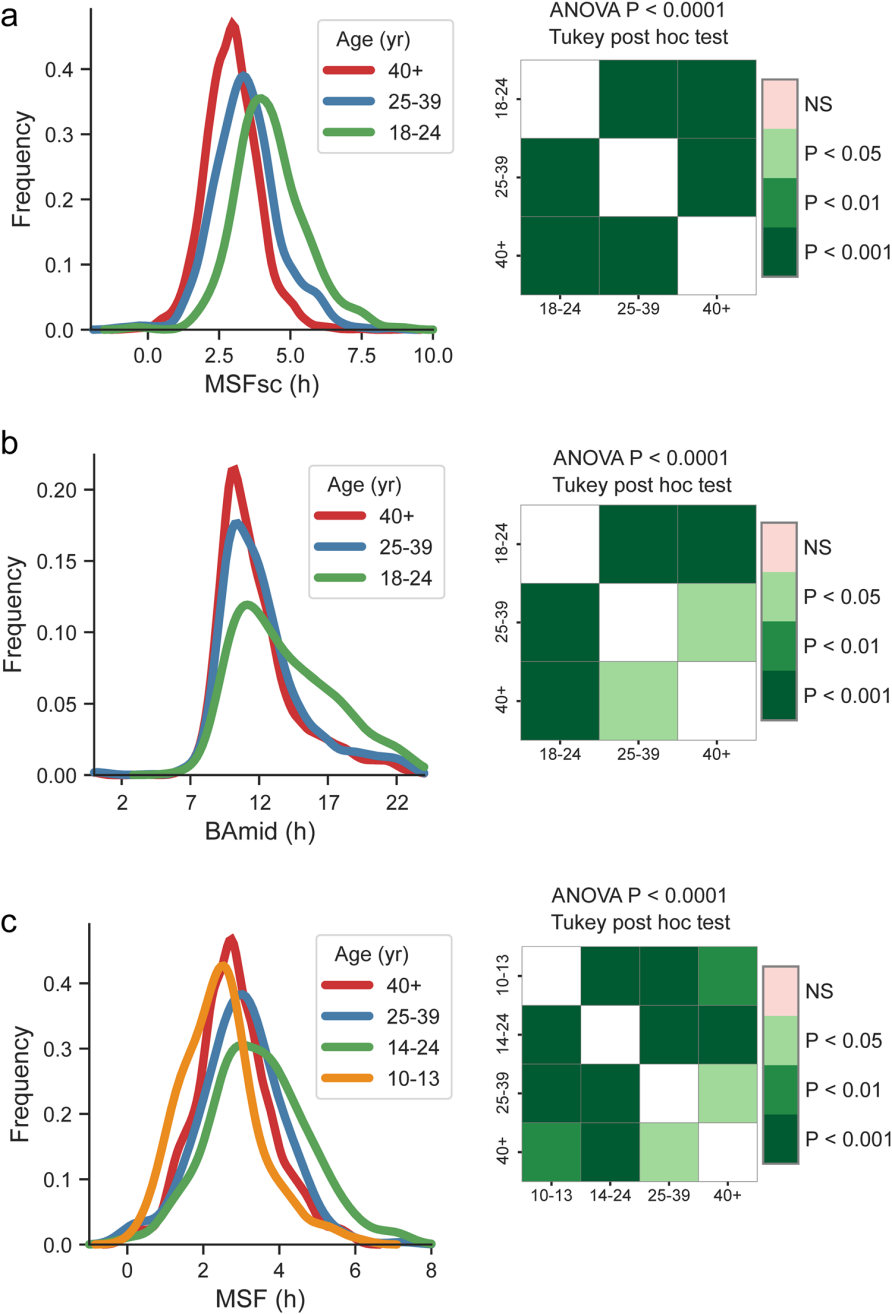
Age and chronotype. Population frequency KDE categorized according to age (yr) of the subjects (green: 18–24 yr old, blue: 25–39 yr old, red: 40 yr and older) for (**a**) MSFsc, (**b**) BAmid, (**c**) MSF. MSF was calculated separately in a Wave 1 survey from time diaries and included children aged 10–13 y (orange) and adolescents aged 14–24 y (green). Heat maps show result of Tukey post hoc multiple comparison test.

For assessment of the association between chronotype and age (Fig. 4), we compared MSFsc and BAmid among 3 age categories of 18+ aged subjects, that is, 18–24, 25–39, and 40+ years old. Data were analysed by one-way ANOVA with Tukey post hoc test. There was a significant age-dependent gradient in MSFsc between the categories (P < 0.0001); group of the oldest subjects (40+ years old) exhibited the earliest MSFsc and group of the adolescents (18–24 years old) exhibited the latest MSFsc (Fig. 4a). Importantly, the same significant gradient was detected also for BAmid (P < 0.0001) (Fig. 4b). Using the data collected by time-use diaries allowed us to involve age category of 10+ years old subjects, who participated in the same survey (for more details, see Methods). Similar method was previously used to assess the association between age and chronotype in the US population^52^. For evaluation of the time-use diary data, we assigned the age categories of 10–13 and 14–24, 25–40 and 40+ year old. For those subjects who described the reported day as being a “typical free day” (n = 1376), we calculated approximate non-adjusted MSF values. In spite of this less precise assessment method, the results confirmed the same significant association between age and MSF values (Fig. 4c) as found for MSFsc and BAmid (shown in Fig. 4a,b). In accordance with the study of Fischer and colleagues^52^, our results also show that the age category of 10–13 years old subjects has significantly earlier MSF and the category of 14–24 year old subjects has significantly later MSF than the other age categories. The data also clearly demonstrate that chronotype in our population sample delays from childhood to the end of adolescence and then progressively advances during adulthood. The same trend has previously been repeatedly reported for chronotype assessed by sleep phase^15,22,53,54^ (reviewed in^9^).

**Figure 5:**
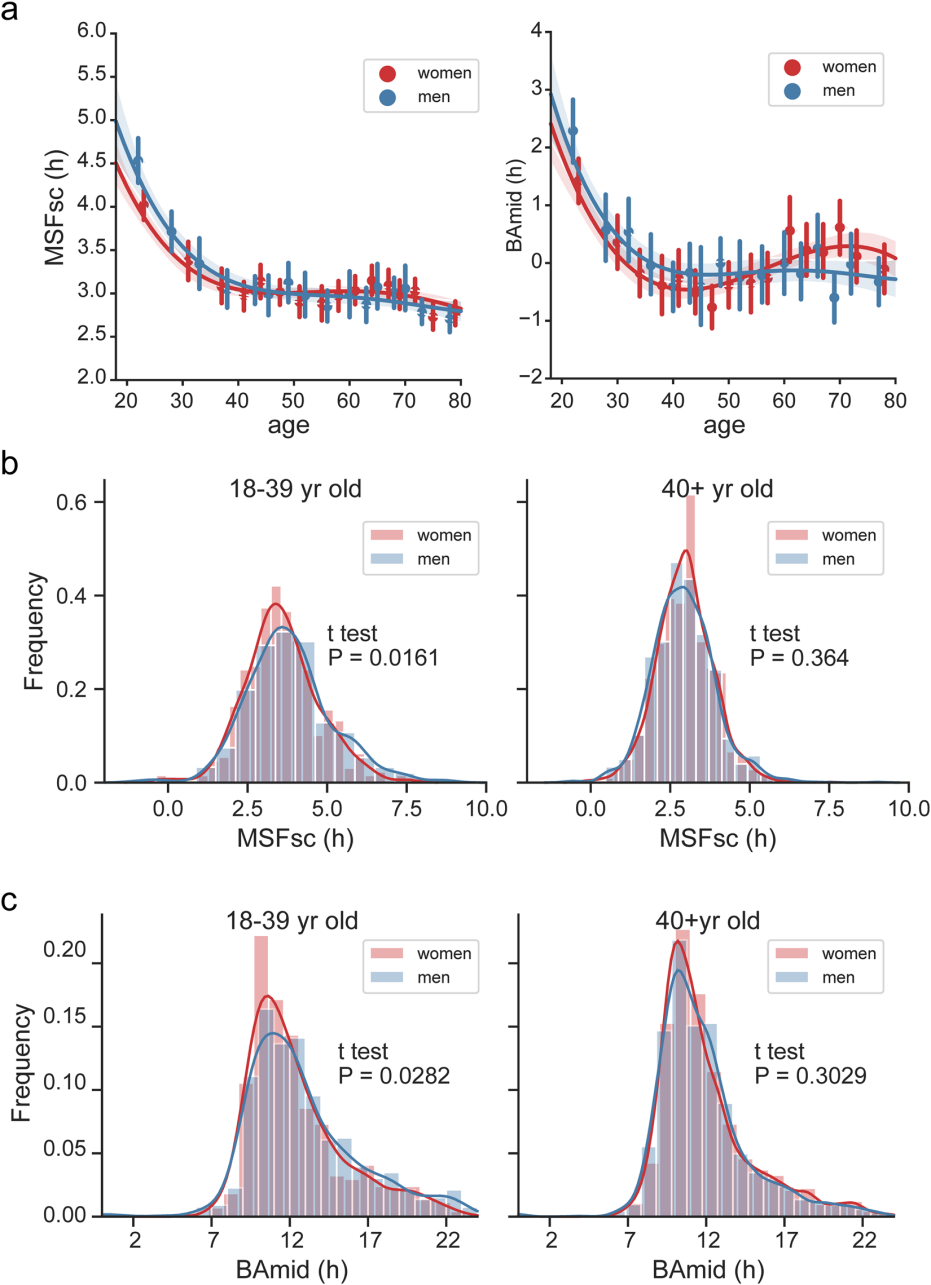
Sex and chronotype. (**a**) MSFsc (left) and BAmid (right) of men (blue) and women (red) was plotted against age of the subject. Means ± SD were calculated in 16 bins, fitted with 4^th^ order polynomial curve and analysed by regression (shading shows a confidence interval computed by inbuilt bootstrap procedure). KDE histograms of men (blue) and women (red) 18–39 yr old (left) or 40+ yr old (right) comparing (**b**) MSFsc, (**c**) BAmid.

We tested association between sex and the age-dependent changes in chronotype for 18+ years old subjects (Fig. 5). When age was not included into the analyses (Fig. 5a), there was no difference in chronotype between men and women assessed by MSFsc (t-test, P = 0.1114), or BAmid (P = 0.4388) (Fig. 5a). Very weak but significant differences between MSFsc of women and men were detectable after age categorization into two groups, ≤39 and 40+ years old (Fig. 5b). Whereas MSFsc was slightly earlier in women than men at the category ≤39 years old (P = 0.0161), the sex difference disappeared for the category 40+ (P = 0.3640). Similarly, the BAmid (Fig. 5c) revealed that women were slightly earlier chronotypes than men for age ≤39 (P = 0.0282) but not for age 40+ (P = 0.3029).

The age-dependent sex difference in chronotype was reported in a recently published meta-analysis data based on MEQ.^55^. Also, a Brazilian cohort study using MEQ demonstrated that women are earlier types to men only up to 30 years old, they do not differ to men between 30–45 years old, and then they become even later types at age 45+ ^28^. The authors speculate about larger plasticity in chronotype changes in women than men. Theoretically, the age-dependent earlier chronotype we found in pre-menopausal women compared to men could be determined by endogenous biological factors, e.g., hormonal, because recently, the levels of sex hormones were associated with chronotype^56^ and sleep quality^57^. Additionally, when assessed at the population level, the peak in chronotype lateness occurring at around the age of 20 has been suggested as a biological marker of the end of adolescence^22^, that is, a developmental stage accompanied with significant hormonal rebuilt. The statement needs to be confirmed in a longitudinal study. Altogether, the results of our study confirmed that chronotype evaluated by both MCTQ and self-assessment exhibits highly significant association with age. The association with sex is significant only for subjects younger than 40 years.

### Social and lifestyle-related factors

**Figure 6:**
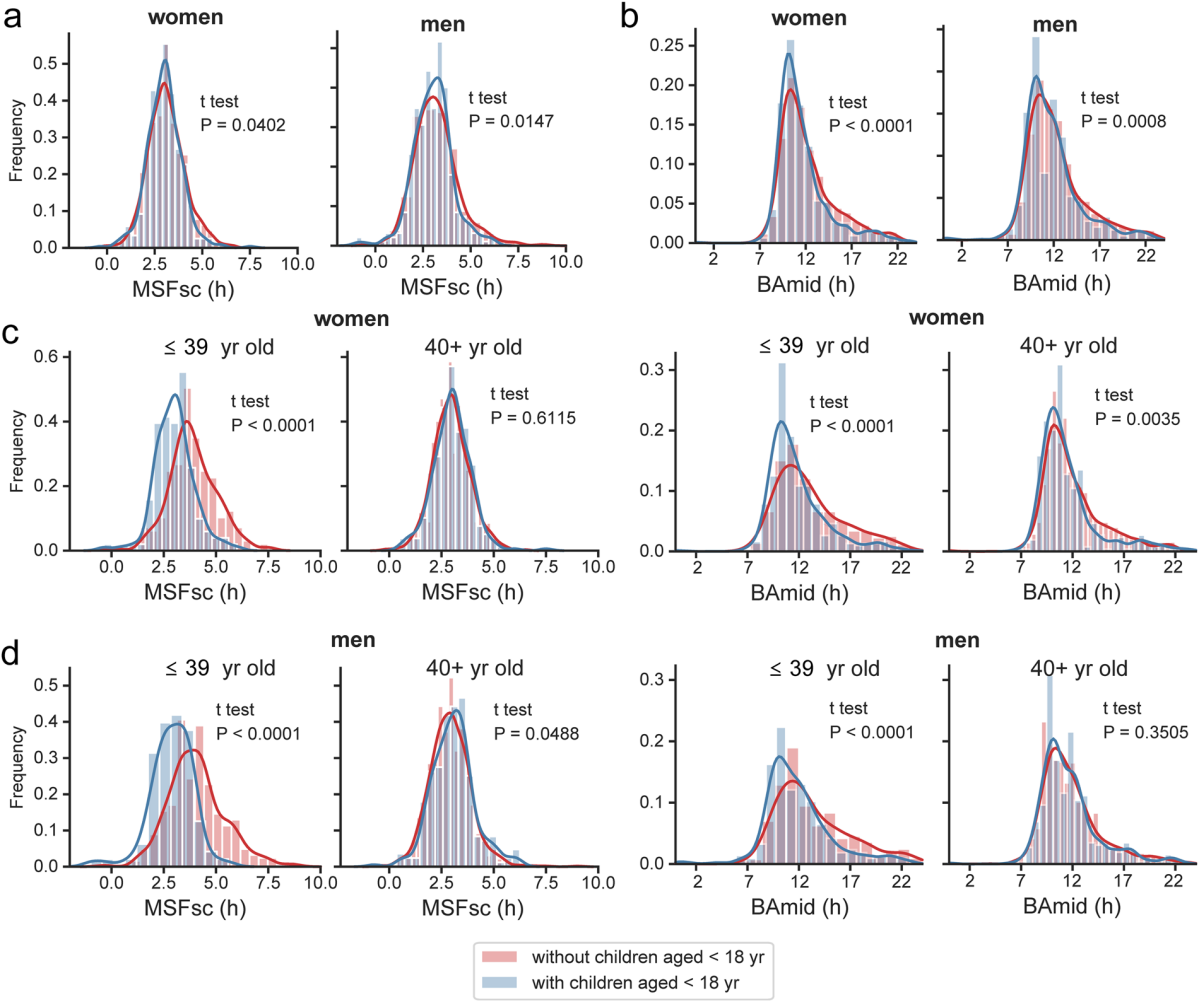
Parenthood and chronotype. (**a**) KDE histograms of MSFsc of adult women (left) and men (right) that either do have (blue) or do not have (red) children aged 0–17 in the same household. (**b**) KDE histograms of BAmid of adult women (left) and men (right) that either do have (blue) or do not have (red) children aged 0–17 in the same household. (**c**) KDE histograms of MSFsc (left) and BAmid (right) of women of two age categories that either do have (blue) or do not have (red) children aged 0–17 in the same household. (**d**) KDE histograms of MSFsc (left) and BAmid (right) of men of two age categories that either do have (blue) or do not have (red) children aged 0–17 in the same household. Student t test was used to compare data coded as the hue of the plot (have vs. do not have children).

We tested a hypothesis that the earlier chronotype we found in women than men at the category ≤39 years old is due to a childcare which might be one of the social factors especially relevant for parenting younger children who typically need more intensive and time-demanding parental care. Therefore, we analysed the association between parenting children (aged up to 17 years) and the chronotype of men and women, either independently of their age, or when divided into two age categories of ≤39 and 40+ years old (Fig. 6). We choose these two age categories based on the assumption that ≤39 years old parents typically raise younger children than 40+ years old parents because the mean parturition age in the Czech Republic is between 28 and 30 years (according to actual statistics of the Czech statistical office, https://www.czso.cz/csu/czso/home). Regardless of the parents’ age categories, the association between childcare and their chronotype was only very weak when assessed by MSFsc (Fig. 6a) for both men (with/without children n = 1170/314; P = 0.0147) and women (with/without children n = 1430/363; P = 0.0402). Nevertheless, the effect was more pronounced for BAmid (Fig. 6b), with significantly earlier chronotype for parentings in both men and women (men with/without children n = 1640/604; P = 0.0008; women with/without children n = 2024/758; P < 0.0001). With differentiation of parents into the age categories, the association between childcare and chronotype became more robust. For women (Fig. 6c), the results revealed a highly significant association of parenting with earlier MSFsc for the age category of ≤39 years old (with/without children n = 127/238; P < 0.0001), but not for the age 40+ (with/without children n = 236/1192; P = 0.6115). The same effect was confirmed by BAmid where again women ≤39 years old with children were significantly earlier types than those who did not take care of children (with/without children n = 366/368; P < 0.0001), and a less pronounced but still significant effect persisted also in 40+ years old women (with/without children n = 1656/392; P = 0.0035). For men (Fig. 6d), a significant parenting effect on earlier chronotype determined by both parameters was significant for the age category of ≤39 years old (MSFsc: with/without children n = 87/283; P < 0.0001; BAmid: with/without children n = 439/220; P < 0.0001). However, there was no effect for 40+ years old men (MSFsc: with/without children n = 227/887; P = 0.0488; BAmid: with/without children n = 1201/384; P = 0. 3505).

The results revealed a significant association between childcare and the parents’ chronotype regardless of their sex if they were younger than 40 years, with a less significant overhang for women older than 40 years. These parents have earlier chronotype than their childless peers. Our results do not support speculation that childcare is the reason for the earlier chronotype in women than men at age younger than 40 year old (see above)^58^ because the association between childcare and chronotype was comparable in both women and men. To our knowledge, this factor has not been systematically correlated with chronotype in previously published work. Therefore, our data revealed for the first time that, at least in the Czech population, childcare is an important social factor, which contributes to modulation of chronotype of men and women at relevant period of life.

**Figure 7:**
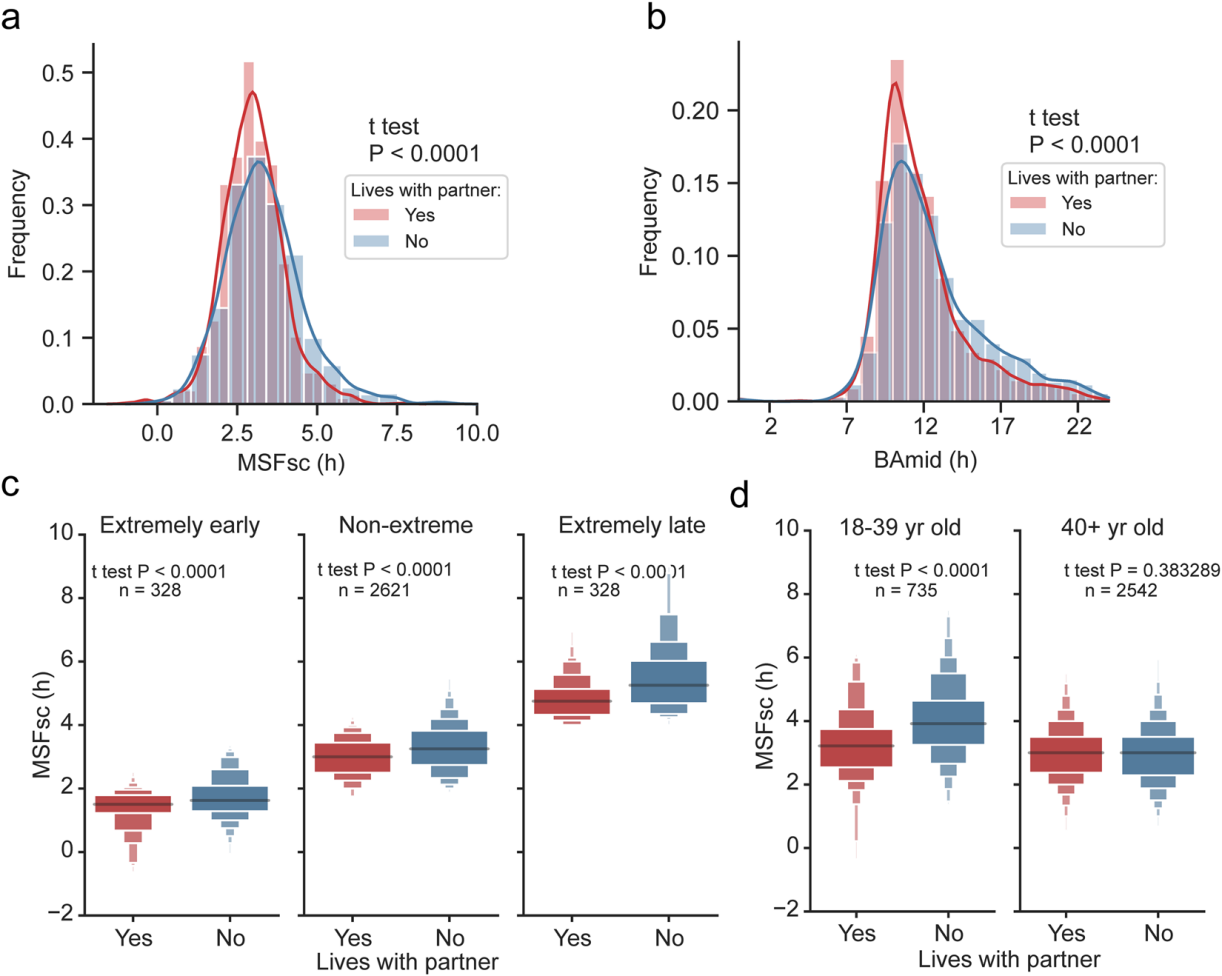
(**a**) KDE histograms of MSFsc of subjects living with a partner (red) compared with those living without a partner (blue) in the same household; (**b**) KDE histograms of BAmid of subjects living with a partner (red) compared with those living without a partner (blue) in the same household. Student t test shows that living with a partner is significantly associated with MSFsc and BAmid of the subject. (**c**) Boxen plot of MSFsc categorized according to presence (yes) or absence (no) of subject’s partner in the same household compared separately for extremely early (left), non-extreme (middle) and extremely late (right) normalized chronotypes. (**d**) Boxen plots of MSFsc categorized according to presence (yes) or absence (no) of subject’s partner in the same household and compared separately for 18–40 years old (left) and older than 40 years (right). Data show significant association of MSFsc with presence of a partner only in younger adults.

Living with a partner is a social factor, which has previously been shown to affect chronotype^59^. Therefore, we compared chronotypes in respondents divided into two groups according to whether or not they shared household with a partner. Subjects who indicated that they live with a partner had significantly earlier chronotype compared to those who lived alone according to both MSFsc (Fig. 7a, p < 0.0001) and BAmid (Fig. 7b, p < 0.0001) analysis. Next, we compared MSFsc between both groups in subjects categorized according to their normalized chronotype (extremely early, extremely late, non-extreme; for categorization details, see Methods) and found that the association between living single and being a later chronotype was highly significant for all three chronotype categories (for P values and n, see Fig. 7c). When age on the subjects was considered, the association of living single with late chronotypes was detectable only in subjects 18–39 years old but not 40+ years old (for P values and n, see Fig. 7d). The results are in accordance with previous studies^59,60^.

**Figure 8:**
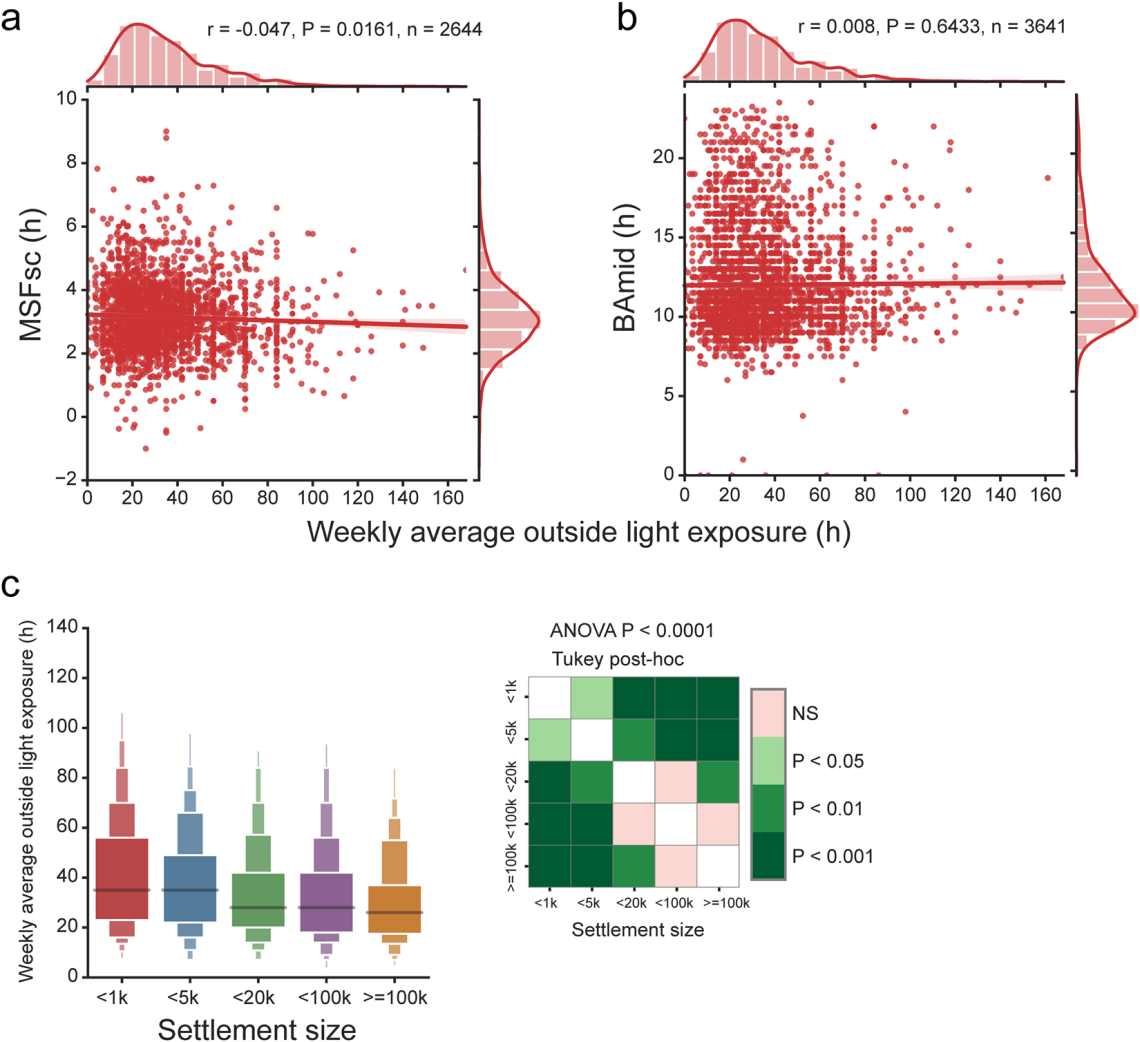
Environmental light exposure and chronotype. (**a**) MSFsc is weakly negatively correlated with time spent outdoors (weekly average in h). (**b**) BAmid does not significantly correlate with time spent outdoors; r and P values show the result of Pearson correlation. (**c**) Boxen plot of weekly average environmental light exposure categorized according to settlement size of subject’s residence. Time spent outdoors is significantly associated with settlement size as shown by one way ANOVA. Heat map shows result of Tukey post hoc multiple comparison test; k is the shorthand for thousands.

Time spent outdoor has previously been recognized as a lifestyle factor which significantly affects chronotype assessed as sleep phase^61^. Individual weekly outdoor light exposure was assessed based on reported typical time spent outside during work and free days, respectively, during the last 3 months before answering the questionnaire. In our population sample we found that weekly average outdoor light exposure was negatively associated with MSFsc (n = 2644, P = 0.0161) (Fig. 8a), but not with BAmid (n = 3641, P = 0.6433) (Fig. 8b). Therefore, we analysed a role of several factors, which may affect time spent outdoor and their effect on chronotype.

First, we tested whether time spent outdoor relates with the settlement size, which we found to have a strong correlation with chronotype in our sample (see Fig. 3). As expected, time spent outdoor had a strong negative association with settlement size (ANOVA P < 0.0001) (Fig. 8c). Therefore, we can speculate that time spent outdoor is a significant factor contributing to the higher prevalence of later chronotypes we found in larger settlements (see Fig. 3 and^61^).

**Figure 9:**
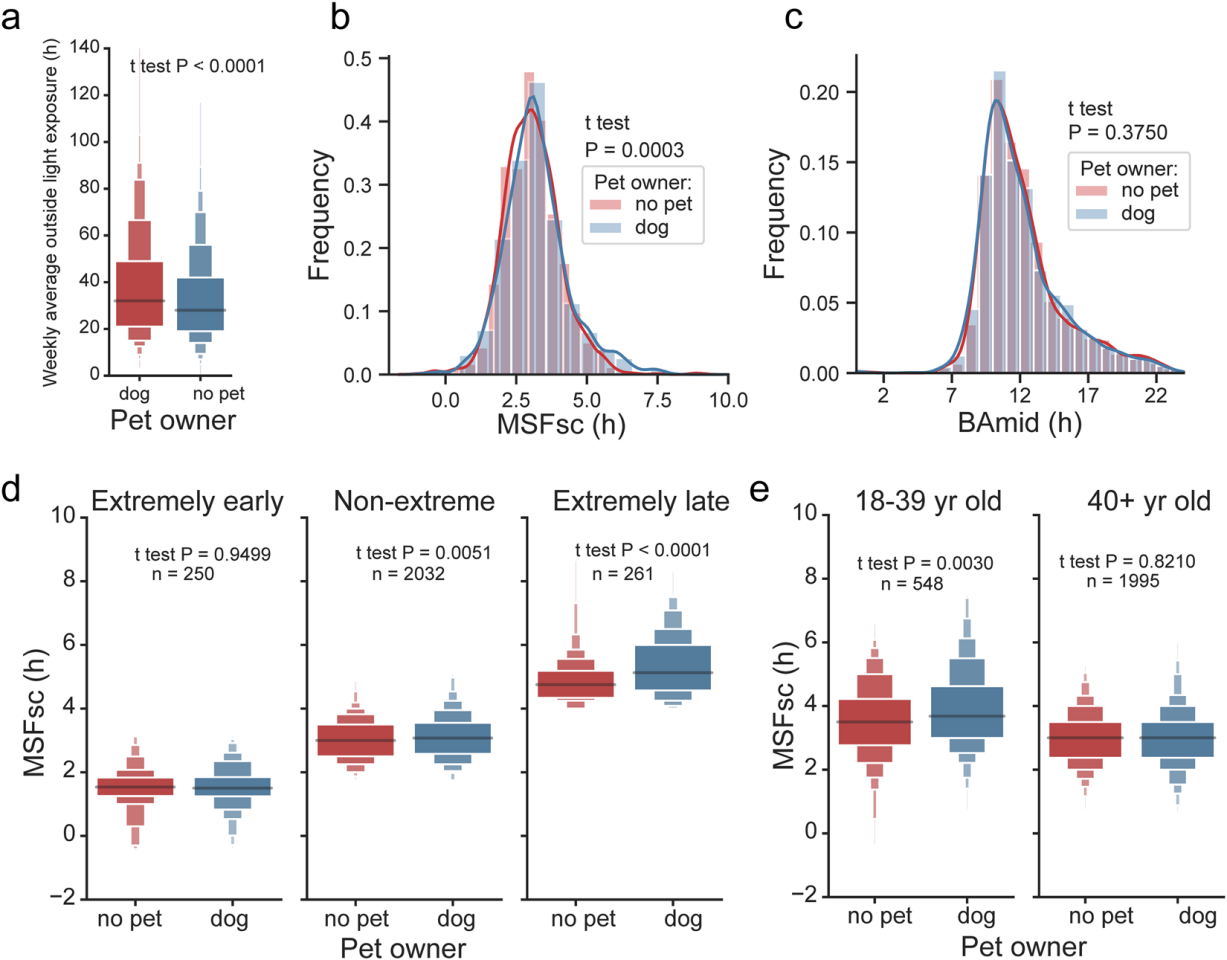
Dog care and chronotype. (**a**) Boxen plot of weekly average time spent outdoors categorized according to subject’s dog ownership. Time spent outdoors is significantly associated with subject’s dog ownership as shown by Student t test. (**b**) KDE histograms of MSFsc of dog owners (blue) compared with those that do not have a pet (red) in the household; (**c**) KDE histograms of BAmid of dog owners (blue) compared with those that do not have a pet (red) in the household. Student t test shows that dog ownership has a significant effect on MSFsc but not on BAmid. (**d**) Boxen plots of MSFsc categorized according to dog ownership and compared separately for extremely early (left), non-extreme (middle) and extremely late (right) normalized chronotypes shows significant association of dog ownership with MSFsc in non-extreme and extremely late, but not extremely early types. (**e**) Boxen plots of MSFsc categorized according to dog ownership and compared separately for subjects 18–39 years old (left) and older than 40 years show significant association of dog ownership with MSFsc only in younger adults.

Next, we identified dog ownership as another lifestyle factor significantly associated with the amount of time spent outdoor (Fig. 9a, with dogs/without pets n = 1282/1551, t-test P < 0.0001). Czech Republic has one of the highest dog/household ratios in the EU (the estimate number of dogs in the Czech households is 2 million per approx. 10 million residents, 41% of households owning at least one dog https://www.statista.com/statistics/515475/dog-ownership-european-union-eu-by-country/). Therefore, we decided to test the link between dog ownership and chronotype in our population sample. We selected dogs from other pets because they need to be walked outdoor, usually early in the morning and late in the evening, providing thus their owners with the exposure to natural daylight especially at the time when light is the most powerful entraining signal. Therefore, we hypothesized that taking care of dogs may potentially keep their owners better synchronized with the solar cycle. Additionally, in employed subjects, the dog walking needs to be done before working hours so it may help to synchronize them with the social time. We compared chronotype (defined by MSFsc and BAmid) of subjects who indicated taking care of dogs and those who had no pets (Fig. 9b). Dog ownership was statistically significantly (t test, P = 0.0003) associated with later chronotype assessed by MSFsc, but not by BAmid (t test, P = 0.3750) (Fig. 9c). The results suggest that whereas the dog care affects sleep phase, the subjectively assessed chronotype is not affected. Testing the dog’s ownership effect on the sleep phase in chronotype-categorized subjects (extremely early, extremely late, non-extreme; for categorization, see Methods) revealed the largest effect in the extremely late chronotypes, weaker effect on non-extreme chronotypes and no effect on extremely early chronotypes (for P values and n, see Fig. 9d). Additionally, the association of dog ownership with the later MSFsc was detectable only in subjects 18–39 years old but not in 40+ old subjects (for P values and n, see Fig. 9e). The number of dog owners was similar for both extremely early and extremely late (Early: have dogs/no pets n = 104/146; Late: have dogs/no pets n = 119/142), suggesting that there is no chronotype-dependent preference in taking care of a dog. Therefore, our data show that dog care is associated with delaying sleep phase selectively in younger subjects who are already significantly later types than older subjects; the early chronotypes seem to be protected against the effect. We may speculate that young subjects with late chronotypes prefer walking dogs late into the night hours to postpone the early morning walking, exposing thus themselves to artificial light at night, which might contribute to their chronotype lengthening. This is in agreement with the hypothesis that young subjects may be able more easily delay their clocks than the older subjects with earlier chronotypes. In opposite, the early types may prefer walking their dogs very early in the morning to avoid the late night walking, which may help keeping them better synchronized with solar/social time. Altogether, the results support our hypothesis that dog care provides better entrainment only to those owners who are early chronotypes, whereas for the late chronotypes the opposite might be true. Specifically, late chronotypes living in lager cities with high level of light pollution might be at a higher risk of postponing their sleep phase even more into the later night hours. We are aware of limitations of these comparisons and speculations. Namely, the data are not corrected for the information whether the dogs needed to be actually walked (which presumably accounts for most dogs living in cities), or whether they were living outdoor in backyards by the house and thus did not need to be walked out (which is more frequent in villages and suburbs). Also, the data are not corrected for other social factors affecting the dog owners and their chronotypes, such as living with a partner or child care. Nevertheless, the results are novel and point at the importance of the lifestyle factor on the sleep schedules.

### Health-related lifestyle and metabolic health parameters

**Figure 10:**
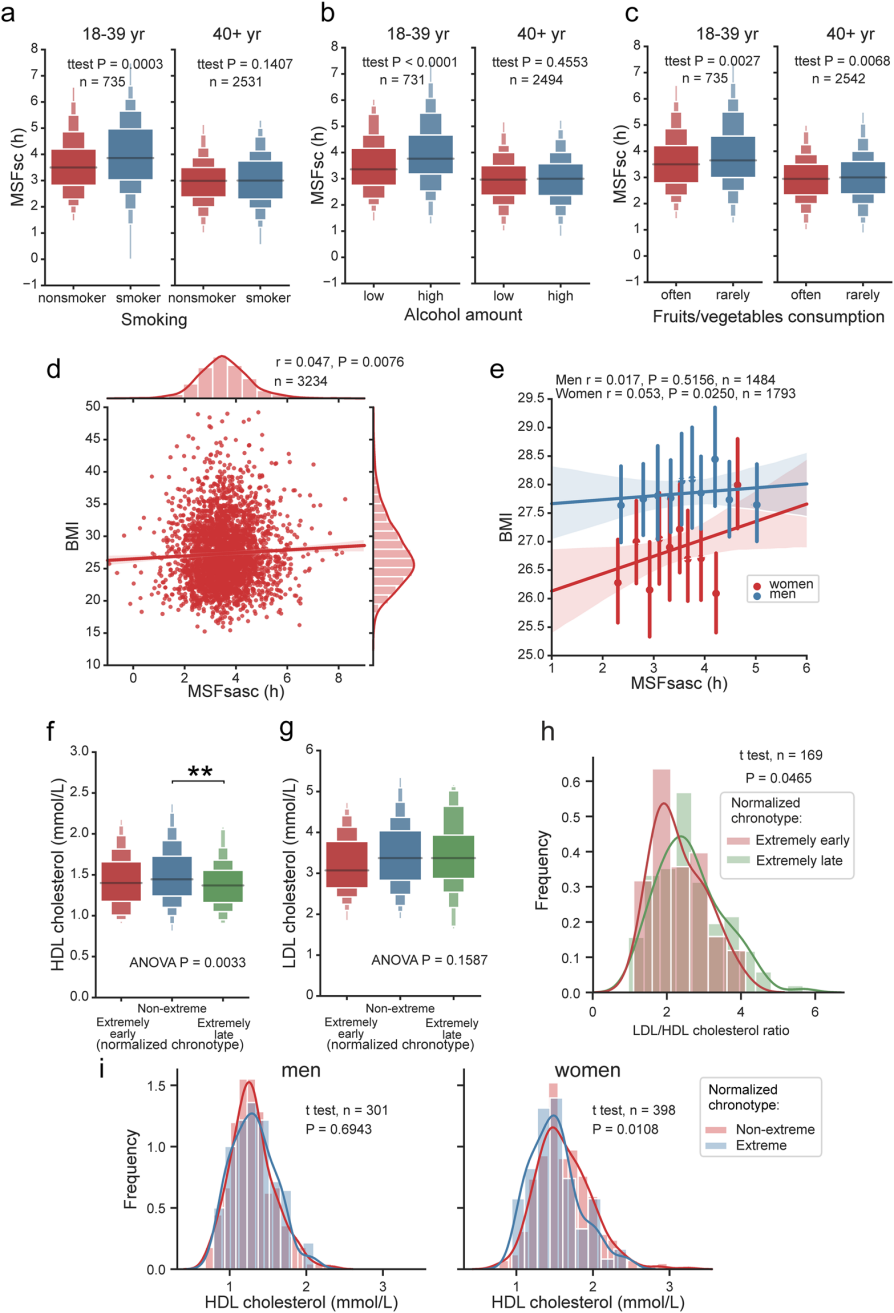
Association of chronotype with health-related lifestyle and health biomarkers. (**a**) Boxen plot of MSFsc categorized according to smoking and compared separately for 18–39 years old (left) and older than 40 years shows significant association of smoking with late MSFsc in younger adults. (**b**) Boxen plot of MSFsc categorized according to amount of consumed alcoholic beverages and compared separately for 18–39 years old (left) and older than 40 years shows significant association of alcohol consumption with late MSFsc in younger adults. (**c**) Boxen plot of MSFsc categorized according to consumption of fruit and vegetables and compared separately for 18–39 years old (left) and older than 40 years shows significant association with MSFsc in both age categories. (**d**) Body mass index (BMI) is significantly correlated with normalized chronotype MSFsasc; r and P values show the result of Pearson correlation. (**e**) BMI of men (blue) and women (red) was plotted against normalized chronotype MSFsasc. Means ± SD were calculated using x_estimator in 10 MSFsasc bins and analysed by linear regression (line with shading denoting a confidence interval computed by inbuilt bootstrap procedure, r and P values show the result of Pearson correlation). BMI is significantly correlated with MSFsasc only in women. Boxen plot of (**f**) HDL cholesterol levels, and (**g**) LDL cholesterol, categorized according to subject’s normalized chronotype. ANOVA shows significant association of late chronotype with lower HDL levels (** designates Tukey post hoc test result, P = 0.0033). (**h**) KDE histograms with t test show comparison of LDL/LDL cholesterol ratio between extremely early (lowest decile, red) and late (highest decile, green) chronotypes. (**i**) KDE histograms of HDL levels of non-extreme (red) chronotypes compared with extreme (lowest and highest decile, blue) chronotypes tested separately for men (left) and women (right). Student t test shows that HDL levels are lower in women but not in men with extreme chronotypes.

We first tested the association between three selected health-related lifestyle factors (smoking, alcohol and fruits/vegetables consumption) and chronotype assessed by sleep phase (MSFsc) of subjects categorized according to age (18–39 and 40+ years old) (for results and statistics, see Fig. 10a–c). Both smoking (Fig. 10a) and increased alcohol consumption (Fig. 10b) were significantly associated with a later MSFsc in 18–39 years old subjects but not in those aged 40 +. These results are in agreement with previously published data^33^. Additionally, we show that less frequent consumption of fruits and vegetables (Fig. 10c) was significantly associated with a later MSFsc in both age categories.

Based on these results, we then tested whether chronotype correlates with physiological parameters of cardio-metabolic health. Since MSFsc is strongly associated with age and weakly with sex (see Figs. 4 and 5), we corrected the values for age and sex and calculated MSFsasc (see Methods for details). This allowed us to correlate chronotype with highly age/sex-dependent health markers, such as body/mass index (BMI), and plasma levels of high density lipoprotein (HDL) and low density lipoprotein (LDL) cholesterol (for statistics see Fig. 10d–i). BMI was positively correlated with MSFsasc (Fig. 10d, P = 0.0076) and categorizing the subjects according to sex revealed that the positive correlation between higher BMI and late chronotype was significant selectively in women (Fig. 10e, P = 0.0250).

Next, participants of Survey II who provided blood samples (n = 1107) were categorized into MSFsasc quantiles as “Extremely early”, “Extremely late” and “Non-extreme” (for details, see Methods). The HDL and LDL levels were compared between the MSFsasc categories by ANOVA and Tukey test. The late chronotypes showed significantly lower levels of cardio-protective HDL (P = 0.0033, Fig. 10f), but no significant difference in LDL levels (P = 0.1587, Fig. 10g). Importantly, the LDL/HDL cholesterol ratio differed between extremely early and late chronotypes (P = 0.0465, Fig. 10h), implying that the cholesterol index is worse in late than early chronotypes. Similarly to BMI, the lower HDL levels were significantly associated in with MSFsasc only in women (for statistics, see Fig. 10i), but not in men (Fig. 10e). Altogether, our results clearly demonstrate that women’s chronotype is more associated with metabolic health than men’s chronotype, with extremely late chronotype being associated with more adverse cardio-metabolic health state. The findings extend previously published data demonstrating association of evening chronotype with increased cardio-metabolic risk^62–65^. Our results are in agreement with a recently published study in young subjects showing association between late chronotype and BMI in female, but not male, adolescents^66^.

## Summary and General Discussion

This study presents the first detailed larger scale chronotyping in the Czech Republic. In agreement with our hypothesis, the data from the complex panel socio-demographic and socio-physiological surveys revealed that the chronotype of the Czech population had an almost normal distribution when defined as mid sleep phase (MSFsc) with the peak being slightly earlier than reported for west-European populations. Chronotype exhibited significant east-to-westward and north-to-southward clines, and was settlement size-dependent. It was associated with age, sex, partnership and time spent outdoor as demonstrated previously. Moreover, for subjects younger than 40 years, childcare was highly associated with earlier chronotype, while dog care was associated with later chronotype. Body mass index correlated with later chronotype in women whose extreme chronotype was also associated with lower plasma levels of protective HDL cholesterol.

Whereas the correlations between chronotype and age, sex, geographic location, and selected social and health factors found in our study are in accordance with the previously published studies, our results provide important information on chronotype distribution within a relatively small geographic region located in the central-to-east part of the CET zone. The information is essential because the decision to abolish the periodic change between ST and DST raises the issue which of the two social times should be maintained yearlong in European countries. The prevalent MSFsc in the population at 3.13 h ± 0.02 h demonstrates that the mean of sleep phase in Czech population is earlier than previously reported for other cohorts^8,9^. This favours yearlong ST as more appropriate for this geographic location for reasons summarized below. Current practice of switching to DST advances social time by 1 h relatively to solar time during the period from April till October. However, leaving it yearlong would extend the duration of the advanced social time into winter season when daylight shortens up to only 8 h per day; on the shortest days on ST, the sun rises at around 8 a.m. and sets at around 4 p.m., but on DST, it would be at 9 a.m. and 5 p.m., respectively. In the Czech Republic, the majority of schools start at 8 am and many occupations even earlier. Many people commute relatively long distances to work or school, which shifts the waking time into much earlier hours. Consequently, with the DST in winter, majority of the population would need to be active for several hours in darkness in the morning during a longer part of the year. Especially late chronotypes need to be exposed to morning light in order to advance their slower clocks. Therefore, the insufficient morning light exposure could preclude proper entrainment of their clocks. Moreover, the reduced daylight exposure would affect also other people with extreme chronotypes whose clocks tend to drift from the 24 h cycle. Additionally, the lack of the daylight may impair mood and cognitive functions with plausible impact on work and school performance^67^.

As already mentioned, in population there are individuals whose chronotype deviates from the average, being earlier or later types. Our study confirmed that women are on average slightly earlier chronotypes than men when they are young, but the difference disappears with their older age. Additionally, we revealed highly significant association between childcare and chronotype in the age-relevant group of parents (both men and women) and found that the chronotype of younger children and their parents is earlier than the average. Therefore, lack of the morning light in winter may affect performance in young children and their economically active parents. Importantly, yearlong DST would also negatively affect clock entrainment of subjects who tend to be later chronotypes. For example, adolescents and people living alone without a partner are more likely later chronotypes. The association is even stronger for people living in larger cities (independent of whether they live alone or not), where, according to our survey, there is a higher frequency of late chronotypes. This is especially important as more and more people are moving from the countryside to larger cities due to better socio-economic conditions. Large cities are characterized by life around the clock, which presents more opportunity for nighttime activities associated with high exposure to artificial light at night. At the same time, urban lifestyle requires that people spend most time indoors, which shields them from sufficient exposure to natural daylight during the day. We found significant negative correlation between settlement size and time spent outdoor. Combination of artificial light at night and lack of daylight results in weaker entrainment of circadian clock. Also, young people with late chronotypes tend to have even more delayed sleep phase if they are exposing themselves to artificial light at night, which according to our study might be associated with walking their dogs. Again, this factor is relevant because it applies to a large proportion of Czech households who own dogs.

Altogether, we can predict that with a reduction of the morning light due to the yearlong DST, most people living in cities and adopting urban lifestyle will be experiencing more difficulties to keep their clocks entrained with the advanced social time, and/or to maintain vigilance and good work/school performance in morning hours. As subjects at the highest risk we identified adolescents, young adults, specifically women, parents and their children, and the group of young dog owners. It is understandable that there is a minority of people in the population who would not have a problem with yearlong DST, such as the extremely late chronotypes who are free to start their activity later during the day. Nevertheless, we can conclude from results of our survey that the yearlong DST would have a predominantly negative impact on majority of the Czech population. The conclusion is important because improper entrainment of circadian clock has been associated with health problems in various studies^62,63^ and our data, showing significant association between extreme chronotype and BMI and lower plasma levels of cardio-protective biomarkers specifically in women, support these associations. Therefore, for the Czech Republic, and likely also for neighbouring countries, maintaining yearlong ST rather than DST would be preferable choice for preventing health problems in majority of the population. This conclusion is in agreement with recently published views and has been extensively discussed elsewhere^36,68^.

## Strength and Limitations

The large scale socio-demographic and health surveys performed at national levels provide the unique opportunity to analyse complex data related to lifestyle of representative population samples. Importantly, such surveys employ native language of the participants, which is especially important for inclusion of all socio-economic groups. The surveys allow analysing population-characteristic features, the significance of which might not be detected in the cross-national surveys. Our study is based on two surveys performed by visits in the randomly selected households, thus avoiding a bias of the internet access, language, or economic and education levels, that might affect the data collected by website questionnaires. Importantly, for most of the analyses we used two independent measures of chronotype (sleep phase and self-assessment) which mostly provided comparable results, confirming validity of methodology used in this study. Another advantage of our study is that we had access to blood samples of group of the chronotyped subjects allowing us to correlate their chronotype and cardio-metabolic biomarkers.

A major limitation of the study is that we cannot compare between the impact of implementation of the yearlong ST and DST on the population chronotype in an exact way. Nevertheless, our results provide the starting point for such possible comparisons to be done in the future, whichever of these situations will be adopted. Another limitation, common to most studies on population chronotyping, is the fact that we cannot provide a causal evidence for the statistically significant associations between chronotype and various social, life-style and health factors. Longitudinal well-controlled studies are needed.

## Data Availability

Materials, data and associated protocols are available to readers.
